# TPGS-Stabilized Curcumin Nanoparticles Exhibit Superior Effect on Carrageenan-Induced Inflammation in Wistar Rat

**DOI:** 10.3390/pharmaceutics8030024

**Published:** 2016-08-16

**Authors:** Heni Rachmawati, Dewi Safitri, Aditya Trias Pradana, I Ketut Adnyana

**Affiliations:** 1 Pharmaceutics Research Group, School of Pharmacy, Bandung Institute of Technology, Bandung 40132, Indonesia; 2 Research Center for Nanosciences and Nanotechnology, Bandung Institute of Technology, Bandung 40132, Indonesia; 3 Pharmacology and Clinical Pharmacy Research Group, School of Pharmacy, Bandung Institute of Technology, Bandung 40132, Indonesia; dewi.s@fa.itb.ac.id (D.S.); ketut@fa.itb.ac.id (I.K.A.); 4 Faculty of Pharmacy, University of Surabaya, Surabaya 60293, Indonesia; aditya_trias@yahoo.com

**Keywords:** curcumin, nanoparticle, inflammation, λ-carrageenan, nanoparticle permeability, Biopharmaceutical Class System (BCS) 4

## Abstract

Curcumin, a hydrophobic polyphenol compound derived from the rhizome of the Curcuma genus, has a wide spectrum of biological and pharmacological applications. Previously, curcumin nanoparticles with different stabilizers had been produced successfully in order to enhance solubility and per oral absorption. In the present study, we tested the anti-inflammatory effect of d-α-Tocopheryl polyethylene glycol 1000 succinate (TPGS)-stabilized curcumin nanoparticles in vivo. Lambda-carrageenan (λ-carrageenan) was used to induce inflammation in rats; it was given by an intraplantar route and intrapelurally through surgery in the pleurisy test. In the λ-carrageenan-induced edema model, TPGS-stabilized curcumin nanoparticles were given orally one hour before induction and at 0.5, 4.5, and 8.5 h after induction with two different doses (1.8 and 0.9 mg/kg body weight (BW)). Sodium diclofenac with a dose of 4.5 mg/kg BW was used as a standard drug. A physical mixture of curcumin-TPGS was also used as a comparison with a higher dose of 60 mg/kg BW. The anti-inflammatory effect was assessed on the edema in the carrageenan-induced paw edema model and by the volume of exudate as well as the number of leukocytes reduced in the pleurisy test. TPGS-stabilized curcumin nanoparticles with lower doses showed better anti-inflammatory effects, indicating the greater absorption capability through the gastrointestinal tract.

## 1. Introduction

Curcumin, commonly called diferuloyl methane, is a hydrophobic natural polyphenolic phytoconstituent derived from the rhizomes of *Curcuma longa Linn* (Zingiberaceae). Curcumin is a major compound of curcuminoid. It has better radical scavenging and antioxidant abilities compared to demethoxycurcumin and bis-demethoxycurcumin. Curcumin is an amphipathic molecule with polar central and flanking regions that are separated by a lipophilic methine segment. It has a pKa1, pKa2, and pKa3 value of 7.8, 8.5, and 9.0, respectively for three acidic protons [1]. Curcumin has a high partition coefficient (log P) in the range of 2.3 to 2.6 [2,3,4]. Despite the polarity of the functional groups and the central dicarbonyl moiety, curcumin, overall, is hydrophobic. This is evidenced by its poor solubility in aqueous solvents and good solubility in organic solvents. Curcumin’s relatively high degree of lipophilicity, due to the methine-rich segments that connect the polar regions, has various important implications on biological activities and also, on the other hand, bears several complexities in the in vitro and in vivo studies [5].

Several reports established ways to improve the solubility of curcumin in aqueous media by increasing the pH. However, changing the conditions to be more alkaline, does not yield a tremendous increase in solubility but does make it much more susceptible to degradation, i.e., it causes alkaline hydrolysis at pH 6.5 due, in part, to the formation of the phenylate anion. So, increasing the pH to dissolve curcumin is not recommended during formulation development. As solubility is one of the two key factors influencing the bioavailability and then biological activity of many active compounds, various efforts are still underway to determine a better method for improving solubility.

Curcumin is a very unique and attractive natural compound due to two contradictive issues: it is a potential therapeutic agent for a wide range of diseases, but problematic in terms of formulation and delivery development.

While the activity of curcumin against a number of disorders is highly promising, the relative bioavailability of curcumin is low [6], reaching only 1% in rats. The absorption process of curcumin is considerably low in the gastrointestinal tract which leads to trace concentrations of curcumin in serum. Ravindranath reported that detectable curcumin was found within 15 min to 20 h after oral administration [7]. Poor oral absorption of curcumin relates to this lower intrinsic activity, lower absorption rate, and high metabolism rate.

A smart, affordable technology that is acceptable for large scale production is required to solve the issues of solubility and permeability. We produced nanoparticles of curcumin by using high pressure homogenization with five distinct stabilizers [8]. A set of physical evaluations demonstrated that polyvinylpyrrolidone (PVP) and TPGS (d-α-Tocopheryl polyethylene glycol 1000 succinate) are the most appropriate stabilizers. This report describes the beneficial aspects of TPGS-stabilized curcumin nanoparticles in treating inflammatory models in animals. Various doses were evaluated to demonstrate the proof of concept on the particle size to solubility correlation. Reducing the particle size down to a nanometer scale was expected to lower the therapeutic dose while increasing the efficacy.

## 2. Materials and Methods

### 2.1. Materials

Curcumin was purchased from PT. Phytochemindo, Reksa, Bogor, Indonesia, d-α-Tocopheryl polyethylene glycol 1000 succinate (TPGS) was commercially obtained from Eastman Chemical Company, Liverpool, UK, diclofenac sodium was purchased from PT Kimia Farma, Bandung, Indonesia, λ-carrageenan was commercially obtained from Sigma-Aldrich, Singapore, NaCl 0.9% was purchased from Otsuka, Tokyo, Japan, Turk’s solution was from Merck, Darmstadt, Germany, heparin was obtained commercially from Inviclot^®^ (Tangerang, Indonesia). All materials used were pharmaceutical grade.

### 2.2. Characterization of Curcumin Nanoparticles

Both conventional curcumin and curcumin nanoparticles were characterized to confirm physical property consistency, including particle size analysis, polidispersity index, zeta potential, morphology with Scanning Electron Microscope (SEM), and curcumin nanoparticle solubility in water.

### 2.3. Particle Size Analysis and Zeta Potential Determination

Analysis on particle size and zeta potential were performed by using the DelsaTMNanoC instrument (Beckman Coulter^®^, Brea, CA, USA). Both curcumin and curcumin nanoparticles were prepared to obtain 100 ppm suspension in water. Before measurement, the suspension was placed in a bath sonicator for approximately 1 min to disrupt any loose-formed agglomerates. Measurement was carried out using particular equipment for particle size and zeta potential analysis.

### 2.4. Solubility Test of Curcumin Nanoparticles

With the same concentration, either curcumin nanoparticles (NC) or conventional curcumin-TPGS were suspended in water. Both samples were placed into an orbital shaker with the following conditions: 25 °C, 100 rpm, for 1 h. Approximately 5 mL of the solution was taken and then centrifuged for 3 min at 12,500 rpm. The samples were filtered using a 0.22 µm filter membrane to separate the insoluble compounds. Curcumin content was determined using spectrophotometric Ultra Violet/Visible (UV/Vis) at wavelength of 425 nm. Water was applied as a standard blank.

### 2.5. Scanning Electron Microscopy

Curcumin and curcumin nanoparticles were fixed on a brass stub and were coated by gold in vacuum conditions. The pictures were taken at an excitation voltage of 10 kV and at 500, 5000, 20,000× magnification by using a JSM-360LA Scanning Microscope (Jeol, Tokyo, Japan).

### 2.6. Fourir Transmission Infrared Spectroscopy (FTIR)

Analysis of Fourir Transmission Infrared Spectroscopy (FTIR) was performed with a Shimadzu FTIR Prestige 21 spectrometer (Shimadzu, Tokyo, Japan) to determine the typical functional groups in curcumin and curcumin nanoparticles. Five milligram samples were prepared with the KBr discs method (160 mg). Wave numbers scanning was conducted with the range of 400–4500 cm^−1^ with resolution of 2 cm^−1^.

### 2.7. X-ray Diffraction

The crystalline phase of both curcumin and PVP-nanocurcumin were determined by X-ray powder diffraction (XPRD) by using a Diano (Woburn, MA, USA) diffractometer with Cu-K′′ radiation (*λ* = 0.1540 nm).

### 2.8. Stability Study

The nanosuspensions were stored in sealed vials at different temperatures (room temperature (RT) and 4 °C) for 30 days. Samples were taken on day 0 (day of production), day 7, and day 30. Characterization was carried out for particle size by Photon Correlation Spectroscopy (PCS), Laser Diffraction (LD), and polarized light microscopy.

### 2.9. Animals

Specific pathogen-free male Wistar rats that were approximately 2 to 3 months of age and 150 to 200 g body weight were used for the activity study, while healthy male Webster mice with 20–30 mg body weight were used for the bio-distribution study. The animals were provided by Animal Laboratory, School Pharmacy, Bandung Institute of Technology, Bandung-Indonesia and were handled under the standard conditions of animal experimentation. Prior to use, all animals were acclimatized for seven days, fed with a standard diet, and allowed to access water ad libitum. The experiment was performed according to the ethical guidelines for investigations in animal laboratory, and approved by The Ethics Committee on Use of Animal Experimentation, School of Pharmacy, Bandung Institute of Technology, Bandung, Indonesia (3 April 2013).

#### 2.9.1. Biodistribution Study of Curcumin Nanoparticle

The bio-distribution study was performed on ^131^I-labeled curcumin nanoparticles given intravenously to healthy male Webster mice (100 μCi/mouse). The bio-distribution of ^131^I-labeled curcumin was observed at 15 min, 1 h and 17 h after intravenous administration of the sample. Blood and major organs were taken and the radioactivity of ^131^I-labeled curcumin was measured on a gamma counter.

#### 2.9.2. Experimental Design for Antiinflammatory Assay of Curcumin Nanoparticle

Two studies were carried out to evaluate the anti-inflammatory effect of TPGS-stabilized curcumin nanoparticles. These studies were differentiated based on the time of treatment: prior and post inflammatory inductions.

In the first study, the animals were divided randomly into five groups: one group was treated with TPGS 1% (control group); one group received sodium diclofenac (SD, 4.5 mg/kg BW), one group received conventional curcumin mixed with TPGS 1% (C, 60 mg/kg BW), one group received TPGS-stabilized curcumin nanoparticles (NC, 1.8 mg/kg BW), and one group received TPGS-stabilized curcumin nanoparticles (NC, 0.9 mg/kg BW). One hour prior to induction, preparation was given orally. Further, 0.05 mL of 1% λ-carrageenan in NaCl 0.9% was injected to induce paw edema via intraplantar. Paw volumes were determined using a mercury plethysmometer every hour for six hours after injection. 

In the second experiment, similar groups were used, but the preparations were given post induction: 30 min, 4.5 h, and 8.5 h. The induction of inflammation was done by injection of 0.05 mL of 1% λ-carrageenan via intraplantar. Observation on paw edema was performed within 10 h after induction.

All studies were performed using one independent experiment with six animals per group.

The inflammatory response is usually quantified by increase in paw volume (edema) which is calculated by the following formula:

(1) $$\%\text{ edema}=\frac{V\text{t}-V\text{o}}{V\text{o}}\times 100\%$$

*V*t = paw’s volume at specific measurement time;
*V*o = basal paw’s volume before induction.

### 2.10. Leukocyte Migration Test (Pleuricy Test)

This measurement was conducted to study the activity of TPGS-stabilized curcumin nanoparticles in a more severe model of inflammation. There were five groups similar to the groups used for the animal study described previously, with similar doses of curcumin and curcumin nanoparticles. One hour prior to induction, the preparations were given orally. To perform this experiment, all rats were anesthetized during surgery by the injection of thiopenthal sodium (50 mg/kg BW, intraperitoneally). Rats were placed on their backs and the hair from the skin over the ribs of the right side was removed using animal clippers. The region was then swabbed with 70% alcohol. A small incision was made into the skin between the seventh and eighth rib. With the intercostal muscle exposed, approximately 0.1 mL of 2% λ-carrageenan suspension was injected into the pleural cavity through this incision. The injection needed to be made swiftly to avoid lung injury. Further, the wound was closed by silk suture 5/0. Twenty-four and forty-eight hours after induction, all rats received a dosage form in accordance to their group. Seventy-two hours after the inflammatory challenge, rats were sacrificed by placing them into a carbon dioxide chamber. Subsequently, the thorax cavity was opened and the exudate collected by aspiration. The exudate was rinsed with 2 mL of NaCl 0.9% that was already mixed with a 5 iu/mL of heparin solution. Approximately 20 µL of collected exudate was then mixed with 380 µL of Turk’s solution to determine the mobilized leukocyte number in the exudate using hemocytometer counting chambers. The results were converted on the basis of exudate volume.

### 2.11. Statistical Evaluation

All data were evaluated using one-way ANOVA followed by Least Significant Difference (LSD) post-hoc analysis by SPSS software version 15 (IBM corp., New York, NY, USA, 2006). The statistical difference was considered significant at *p* < 0.05.

## 3. Results

### 3.1. Characterization of Nanoparticles

#### 3.1.1. Particle Size Analysis, Zeta Potential

The size, size distribution, and surface charge of the samples are presented in Table 1.

#### 3.1.2. Solubility Test

**Figure 1:**
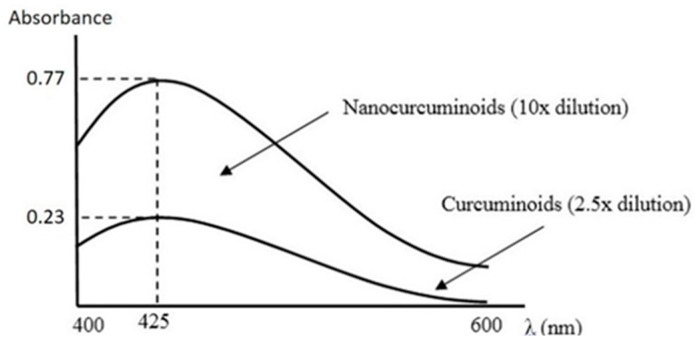
Visible spectra of curcumin and curcumin nanoparticles in water at 25 °C.

The profile of solubility of both curcumin-TPGS and TPGS-stabilized curcumin nanoparticles was reflected by UV/Vis spectra (Figure 1). Simply, by considering the absorbance value, the potency of nanonization is clearly indicated. As seen, the absorbance of curcumin nanoparticles at 425 nm was approximately 13 times higher than conventional curcumin.

#### 3.1.3. Scanning Electron Microscope

**Figure 2:**
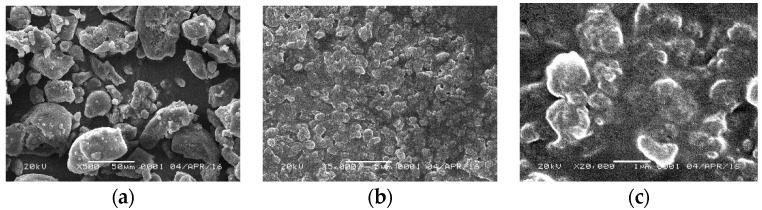
Scanning Electron Microscopic analysis. (**a**) Curcumin 500×; (**b**) curcumin nanoparticles 5000×; (**c**) curcumin nanoparticles 20,000×.

The morphology of both conventional curcumin and curcumin nanoparticles is depicted in Figure 2.

As shown in the Figure 2, the morphology of TPGS-stabilized curcumin nanoparticle showed a more spherical shape as compared with curcumin powder with irregular shapes. Particle size of the nanoparticles was drastically decreased from microscale to nanoscale (~400 nm).

#### 3.1.4. Fourir-Transmission Infrared Spectroscopy

**Figure 3:**
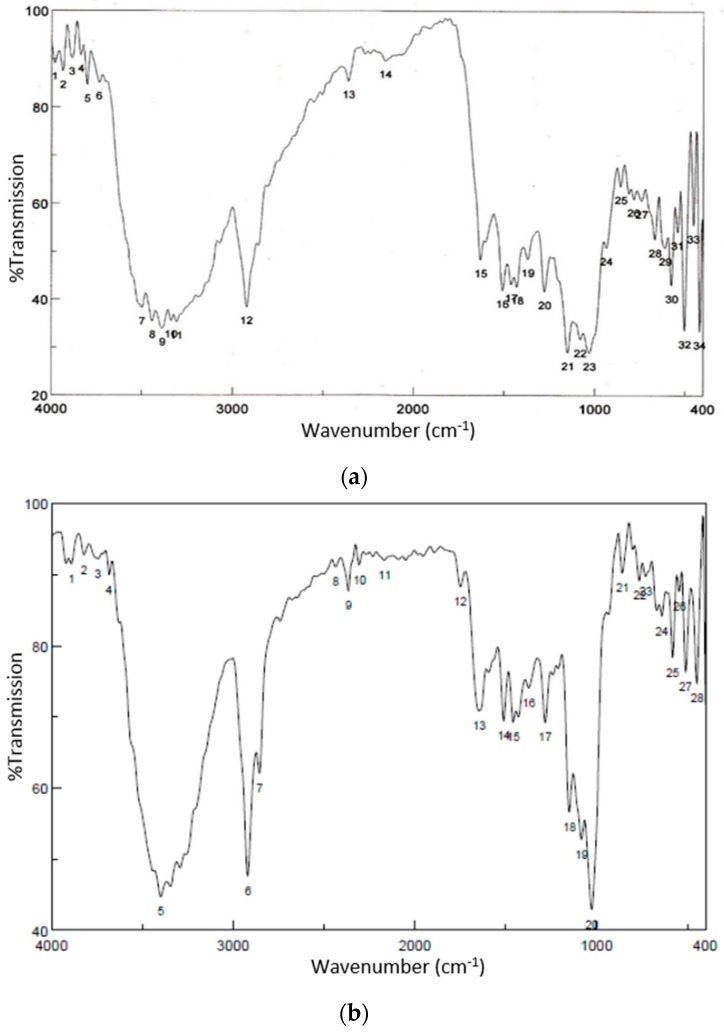
Fourir Transmission Infrared Spectroscopy (FTIR) spectra of curcumin (**a**) and curcumin nanoparticles (**b**).

Figure 3 shows the FTIR spectra of both curcumin and curcumin nanoparticles.

It is clearly shown that the FTIR profile of curcumin is similar to curcumin after the application of high pressure to reduce the particle size of curcumin down to nanometers, except the intensity in particular on peaks at wavenumber 3000–4000 and around 1000.

#### 3.1.5. X-ray Diffarction

**Figure 4:**
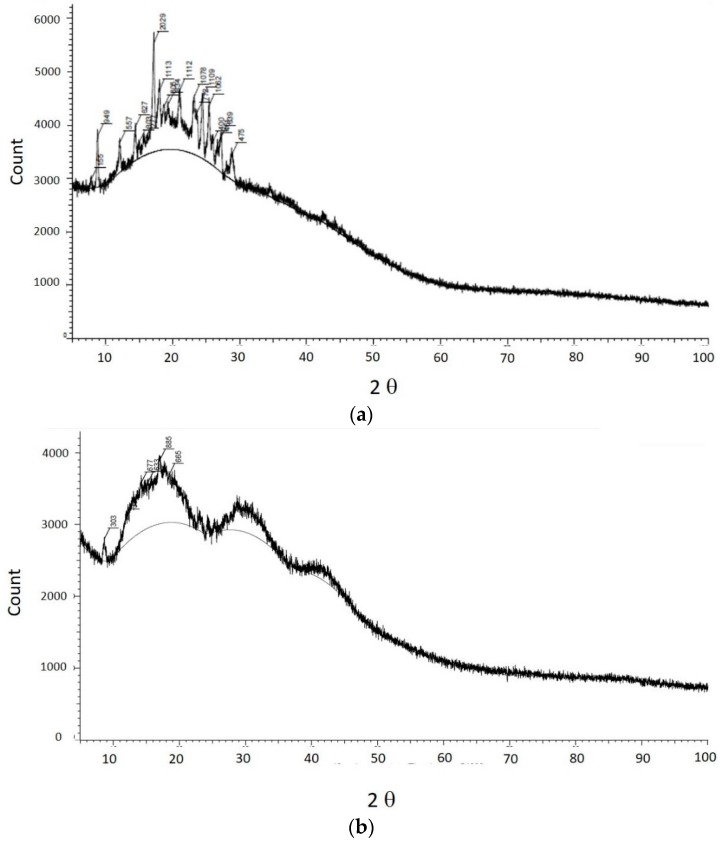
The X-ray diffractograms of curcumin (**a**) and curcumin nanoparticles (**b**).

The crystalinity of curcumin after the high pressure homogenization application to form nanoparticles was analysed using X-ray diffractometry. A reduction in the degree of crystalinity of curcumin nanoparticles was observed (Figure 4). In addition, no other peaks were shown on curcumin nanoparticles. This indicates that the mechanical stress applied during naoparticle production did not result in a new structure.

#### 3.1.6. Stability Study

**Figure 5:**
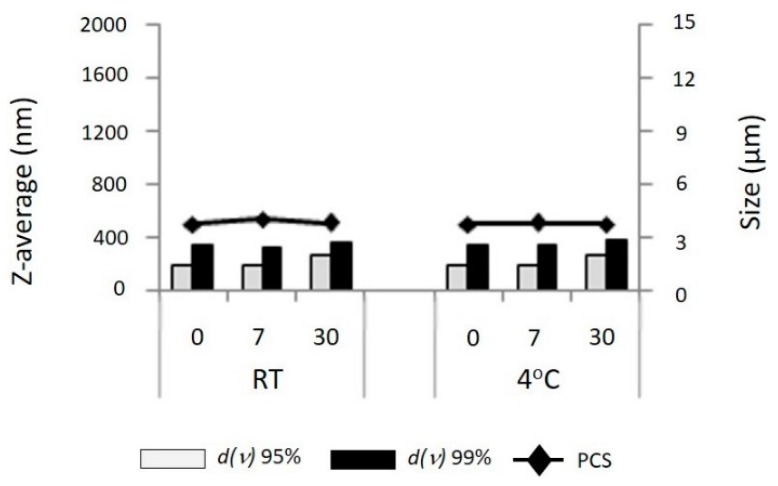
Photon Correlation Spectroscopy (PCS) diameter and Laser Diffraction (LD) diameter *d*(*v*)95% and *d*(*v*)99% of α-Tocopheryl polyethylene glycol 1000 succinate (TPGS)-stabilized curcumin nanosuspensions as a function of days (0–30) stored at room temperature (RT, **left**) and at 4 °C (**right**).

Figure 5 shows the particle size of curcumin nanoparticles after storage for 30 days at 4 °C and room temperature. There was no particle aggregation or crystal growth observed at either storage condition as well as no change in particle distribution size indicated by polidispersity indexed from day 0 to day 30.

**Figure 6:**
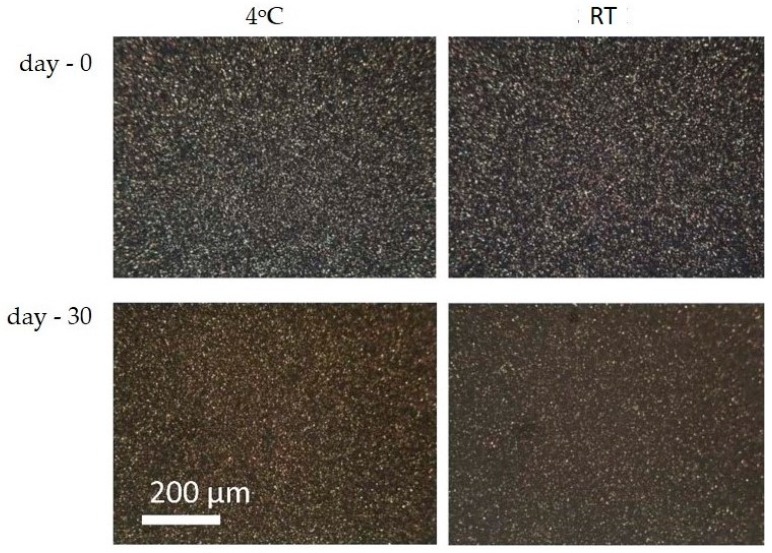
Polarized light micrographs (magnification 160×) of the nanosuspensions after 30 days of storage at 4 °C and room temperature (RT). The scale bars are 200 µm.

The stability data shown in Figure 5 was confirmed by microscopic evaluation on the sample using a polarized microscope (Figure 6).

#### 3.1.7. Biodistribution Study of Curcumin Nanoparticles

**Figure 7:**
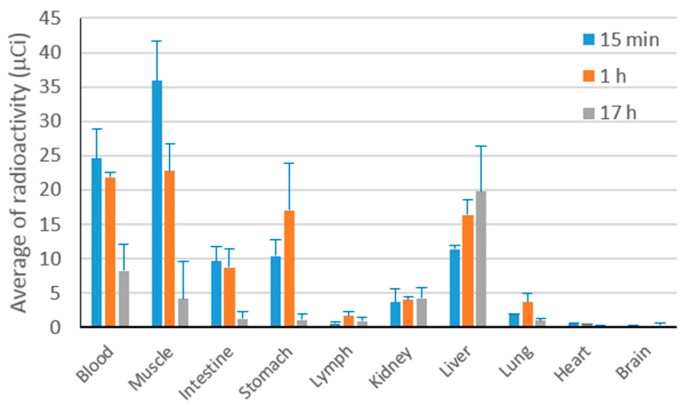
The accumulation profile of ^131^I-labeled curcumin after intravenous administration to male healthy Webster mice.

The in vivo accumulation of curcumin at 15 min, 1 h, and 17 h after intravenous administration of ^131^I-labeled curcumin solution to Webster mice (*n* = 6) is presented in Figure 7.

In the first hour of observation, curcumin was mostly circulated in the blood which is available for pharmacological effect. The distribution of curcumin to kidney, lymph, lung, heart and brain was considered absent (<5%) even at the beginning phase after the preparation entered the body. The liver seems to be the main organ for curcumin metabolism.

### 3.2. Preventive Effect of Curcumin Nanoparticle on λ-Carageenan-Induced Inflammation in Rats

**Figure 8:**
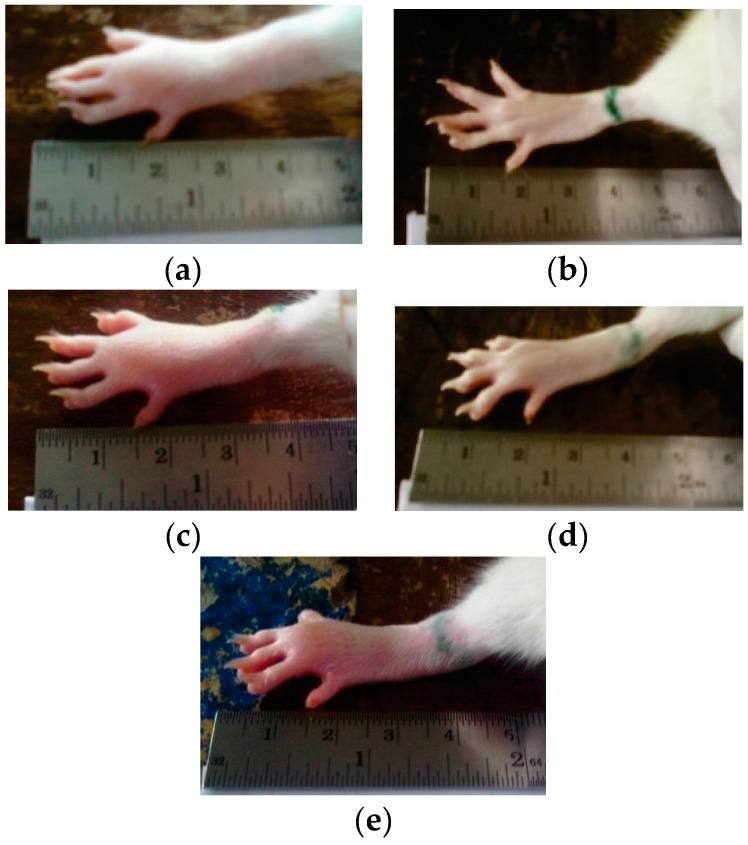
The paw volume observation at 6 h after induction: (**a**) Control group; (**b**) sodium diclofenac (SD) 4.5 mg/kg body weight (BW); (**c**) curcumin (C) 60 mg/kg BW; (**d**) nanocurcumin (NC) 0.9 mg/kg BW; (**e**) NC 1.8 mg/kg BW.

**Figure 9:**
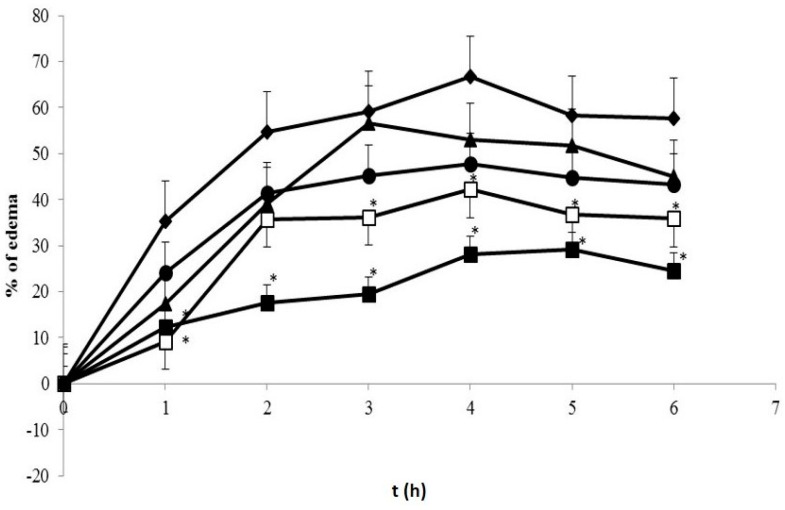
Percentage of paw edema of treated groups 1 h prior to induction. (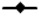) Control group; (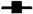) SD 4.5 mg/kg BW; (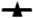) C 60 mg/kg BW; (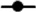) NC 0.9 mg/kg BW; (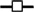) NC 1.8 mg/kg BW. * Indicates statistically different than control group (*p* < 0.05). Each group consisted of six rats.

Figure 8 presents the visual observation on λ-carrageenan-induced edema development on the rats’ paws after treatment with both curcumin and curcumin nanoparticles 6 h after induction. The quantitative analysis on percentage of edema as depicted in Figure 9 shows significant effects only when the animals were treated using curcumin nanoparticles with a dose of 1.8 mg/kg BW (30 times lower than conventional curcumin).

We observed statistically significant differences between the groups that received curcumin nanoparticles (1.8 mg/kg) and the control group at hours 1, 3, 4, 5, and 6 with the percentage of edema 9.22%, 36.23%, 42.28%, 36.71%, and 35.84%, respectively. The percentage of edema indicates the level of inflammation. The lower the percentage reflects the regression of inflammation. Therefore, the treatment which lowers this percentage compared to the control is considerably expressed as an anti-inflammatory effect.

### 3.3. Antiinflammatory Effect of Curcumin Nanoparticle on λ-Carageenan-Induced Inflammation in Rats

**Figure 10:**
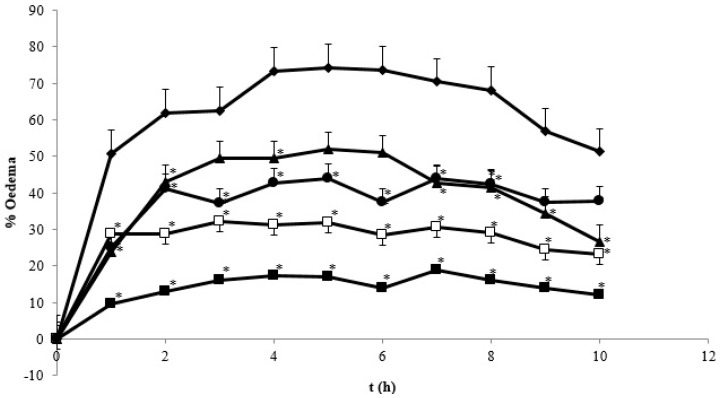
The percentage of paw edema on treated λ-carrageenan-induced rats. (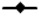) control group; (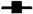) SD 4.5 mg/kg BW treated group; (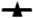) C 60 mg/kg BW group; (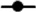) NC 0.9 mg/kg BW group; and (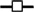) NC 1.8 mg/kg BW. * Indicates statistically different than control group (*p* < 0.05). Each group consisted of six rats.

Figure 10 represents the effect of curcumin after induction on the regression of edema volume in λ-carrageenan-induced rats. As shown, the standard drug sodium diclofenac showed the strongest effect reaching 10.12% at hour 10. The anti-inflammatory effect of curcumin was also displayed in a dose-dependent manner. Reduction of the particle size of curcumin demonstrated a superior effect as reflected by the decreased therapeutic dose. Curcumin in the nanometer scale (~400 nm) required a dose of 60 times less than the conventional curcumin dose to exhibit similar biological effects. The strongest effect of sodium diclofenac is indeed due to the higher dose as compared to curcumin nanoparticles (4.5 versus 1.8 mg/kg BW). We speculate that by applying a similar dose of curcumin nanoparticles to sodium diclofenac, the anti-inflammatory effect would be comparable.

### 3.4. Pleurisy Test

Pleurisy is a well-known phenomenon of exudative inflammation in living organisms. Carrageenan-induced pleurisy in rats is considered to be a suitable inflammatory model in which fluid extravasation, leukocyte migration, and the various biochemical parameters can be measured in the exudate [9]. The higher values of such parameters indicate the severity of inflammation. Thus, compounds that are capable of reducing exudate formation, suppressing leukocyte migration, as well as improving biochemical properties can be proven as anti-inflammatory agents.

Table 2 presents the anti-inflammatory effect of curcumin nanoparticles in terms of the reduction in the number of leukocytes and exudate formation. A similar trend of anti-inflammatory effects to Figure 6 was also revealed. Sodium diclofenac 4.5 mg/kg BW as a standard drug demonstrated the strongest effect.

## 4. Discussion

We successfully developed curcumin nanoparticles and completed a physical characterization [5]. The physical characterization of TPGS-stabilized curcumin nanoparticles such as FTIR, SEM, and X-ray Diffraction (XRD) as well as a month stability test reported here confirmed the function of TPGS as an appropriate stabilizer preventing curcumin nanoparticle agglomeration. The FTIR data suggest no structure alteration after high pressure homogenization was applied on curcumin to form nanoparticles. These data are important in regard to the biological activity of curcumin described in this report. While the XRD data suggest any reduction on the curcumin nanoparticle crystallinity may also contribute slightly to a curcumin solubility enhancement in addition to particle size reduction. Curcumin is a unique, pure bioactive compound. Curcumin is a main phenolic constituent in the Curcuma genus, which is broadly known to exhibit a wide range of pharmacological effects: anti-inflammation, antibacterial, antioxidant agent, and has been widely used for treating Alzheimer’s disease (AD), Parkinson’s disease, multiple sclerosis, epilepsy, cerebral injury, cardiovascular diseases (CVDs), cancer, allergy, asthma, bronchitis, colitis, rheumatoid arthritis, renal ischemia, psoriasis, diabetes, obesity, depression, fatigue, and acquired immune deficiency syndrome (AIDS) [10]. In particular as an anti-inflammatory, curcumin is noticeably effective both in acute and chronic inflammations as described in this report.

λ-Carrageenan used in this study, is a complex group of polysaccharides made up of repeating galactose monomers. The inflammation induced by carrageenan is considerably acute, and not only causes edema, but also hyperalgesia and erythema resulting from the action of pro-inflammatory agents such as bradykinin, histamine, tachykinins, complement and reactive oxygen, and nitrogen species [11]. Through these cascades, swollen paws due to fluid extravasation into the injection site is a clear visual manifestation, therefore, acting as a good parameter to screen the anti-inflammatory drugs. The progression of paw edema is also triggered by neutrophils which migrate to the site of injection and generate pro-inflammatory reactive oxygen as well as other species. We challenged our developed curcumin nanoparticle in different inflammatory models as most of the natural compounds have a weak therapeutic effect and require high therapeutic doses especially in severe diseases. We tested our approach to observe whether particle size reduction on curcumin down to ~400 nm followed by the presence of TPGS decreased the therapeutic dose of curcumin significantly in the model of chronic inflammation.

In the case of curcumin as an anti-inflammatory agent, the compound interacts with numerous molecular targets involved in the progression of inflammation. As reported previously, this interaction mediates curcumin to down-regulate various pro-inflammatory cytokines, which include interrupting formation of prostaglandin, inhibiting cyclooxygenase (COX), lipoxygenase, inducible Nitric Oxide Synthase (iNOS), and other pro-inflammatory mediators such as tumor necrosis factor (TNF-α), interleukins (IL-1, IL-2, IL-6, IL-8, IL-12) and chemokines [12,13,14,15]. As described by Heger et al. [16], curcumin has seven different chemical properties facilitating intermolecular interactions hence association with its biomolecular targets. These include (1) H-bond donating and accepting capacity of the b-dicarbonyl moiety; (2) H-bond accepting and donating capacity of the phenylic hydroxyl residues; (3) H-bond accepting capacity of the ether residue in the methoxy groups; (4) multivalent metal and nonmetal cation binding properties; (5) high partition coefficient (log P); (6) rotamerization around multiple C–C bonds; and (7) acceptor of a Michael reaction. In line with the report of Jeong et al., α,β-unsaturated diketone moiety of curcumin is a Michael reaction acceptor which is a major class of phase-II enzyme inducers [17]. This property is suggested to be responsible for stimulating HO-1 and NF-κB suppression in cells by curcumin. The anti-inflammatory effect of both curcumin and curcumin nanoparticles reported here is suggested through those mechanisms.

The more potent anti-inflammatory effect of curcumin nanoparticles as seen in Figure 8, Figure 9 and Figure 10 is clearly via its improved solubility. The particle size is the initial determining factor of curcumin bioavailability. Curcumin is a Biopharmaceutical Class System (BCS) 4 compound which means the solubility and permeability are problematic and influence its efficacy. Reducing the particle size of curcumin down to nanometer scale (~400 nm) as described here, enhanced its saturated solubility by 13 times (Figure 1). We suggest that the particle size is a key factor determining the ability of curcumin to cross the gut membrane. The presence of TPGS in nanoparticle formula is also considered to influence the therapeutic effect of curcumin nanoparticles which in turn effects the therapeutic dose. TPGS (d-α-Tocopheryl polyethylene glycol 1000 succinate) is a surfactant but also an absorption enhancer through P-gp inhibition. The presence of TPGS in curcumin nanoparticles provides a substantial improvement to curcumin absorption. Thus, reducing particle size of curcumin down to ~400 nm combined with TPGS, as reported here, clearly increases the potency of curcumin by 30 times compared to conventional curcumin. This increase is comparable to a standard anti-inflammatory drug sodium diclofenac. Increased bioavailability was due to a combination of increased surface area-to-volume ratio, possible micelle formation, and enhanced permeability. There is a clear relationship between the particle size and absorption enhancement with the therapeutic dose of BCS 4 drugs, including curcumin. As seen in Figure 1 and Figure 2c, the solubility of curcumin-TPGS is far lower leading to less efficacy as compared to TPGS-stabilized curcumin nanoparticle. Clearly, improved solubility is an important initial step before the action of absorption enhancer TPGS can start. Our TPGS-stabilized curcumin nanoparticles are, therefore, promising as an alternative therapy, in particular for chronic inflammatory diseases. Well known serious side effects of frequently used oral non steroid anti-inflammatory drugs (NSAIDs) are still an issue for successful treatment in chronic inflammation.

## 5. Conclusions

Curcumin nanoparticles demonstrate superior anti-inflammatory effects in an animal model of acute and chronic inflammations, when given orally. It is suggested that by reducing the particle size of curcumin to nanoscale, the bio-availability is improved through enhancing the solubility. As curcumin is an active compound in the classification of BCS 4, solubility is not the only limiting factor influencing the activity, but also the intestinal permeability. TPGS-stabilized curcumin nanoparticles offer a solution for both absorption barriers. The benefit of improving bio-availability through nanotechnology is in minimizing the therapeutic dose as proven in this study. The issue of drug toxicity is, further, an important target in addition to efficacy, when the therapeutic dose of such drugs is decreased. So, the potency of the natural compound in therapy improved significantly by nanonization.

## Figures and Tables

**Table 1:** Physical characteristics of curcumin and curcumin nanoparticles.

Parameters	Curcumin	Curcumin Nanoparticles
Particle size (nm)	2273.6	435.7
Polydispersity index	0.667	0.316
Zeta potential (mV)	−13.60	−21.20

**Table 2:** The effects of treatments on the pleurisy test.

Group (N = 6)	Volume of Exudate (mL)	The Number of Leukocytes (× 10^8^/mL)
Control	4.80 ± 0.80	5.65 ± 1.77
SD 4.5 mg/kg BW	2.18 ± 0.21 ^a^	1.36 ± 0.12 ^a^
C 60 MG/KG BW	3.25 ± 0.29 ^ab^	2.89 ± 1.05 ^ab^
NC 0.9 mg/kg BW	3.25 ± 0.82 ^ab^	2.34 ± 1.16 ^a^
NC 1.8 mg/kg BW	2.95 ± 0.63 ^ab^	1.73 ± 1.08 ^a^

The values represent mean ± standard deviation. ^a^: Significantly different than the control group (*p* < 0.05); ^ab^: significantly different than the sodium diclofenac-treated group (*p* < 0.05). SD: sodium diclofenac; C: curcumin; NC: nanocurcumin.
